# Supplementary figures and images for: The PagKNAT2/6b-PagBOP1/2a Regulatory Module Controls Leaf Morphogenesis in Populus
Source: Int J Mol Sci. 2022 May 17;23(10):5581. doi: 10.3390/ijms23105581 (PMC9145908; doi:10.3390/ijms23105581)

Supplementary Figures

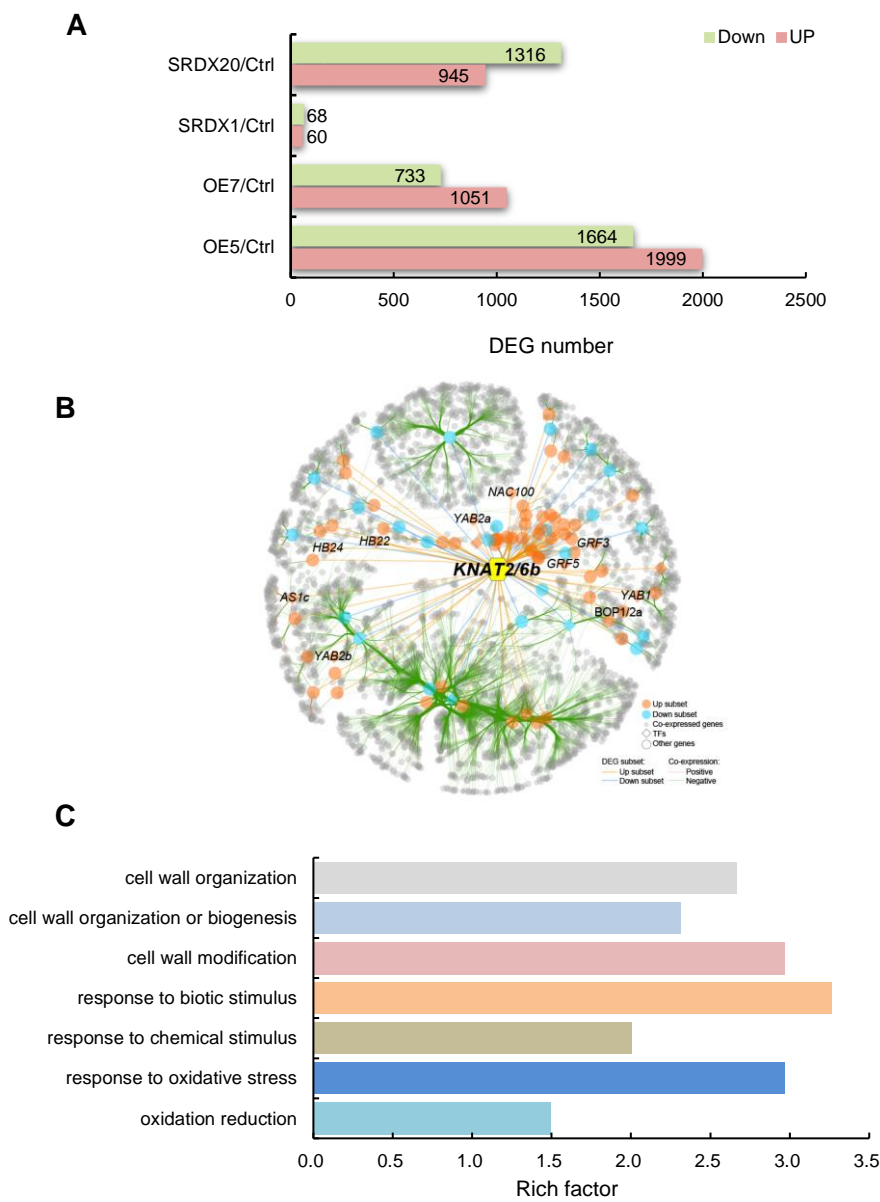

Figure S1

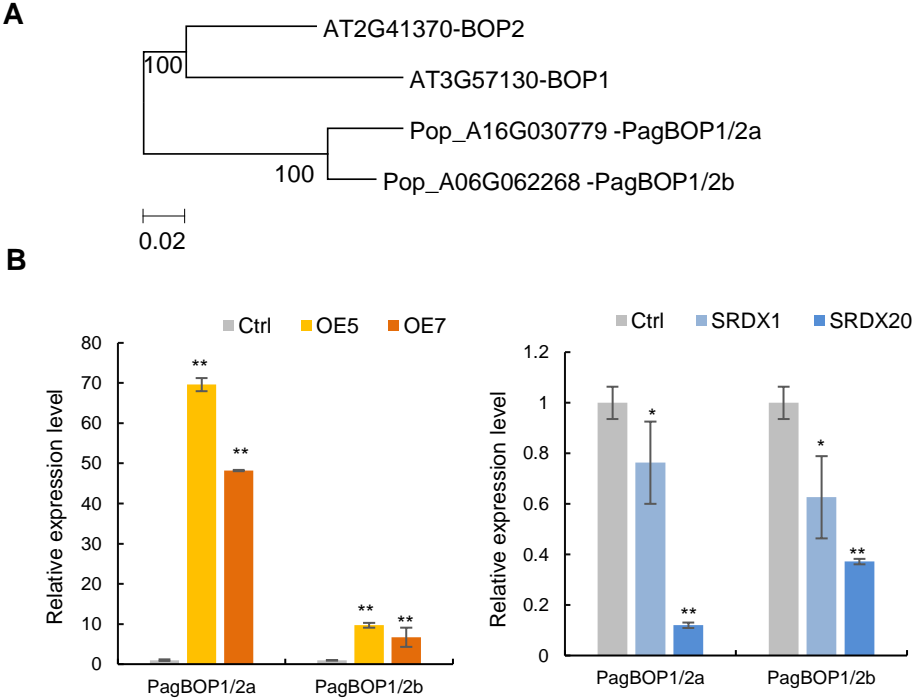

Figure S2

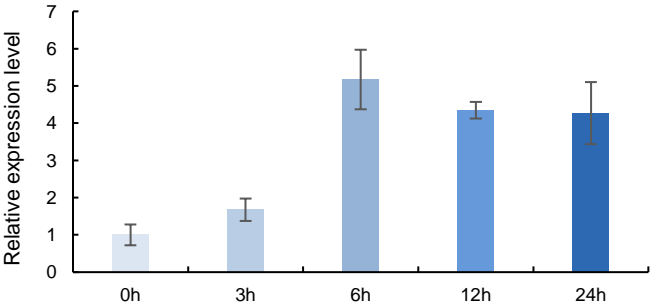

Figure S3

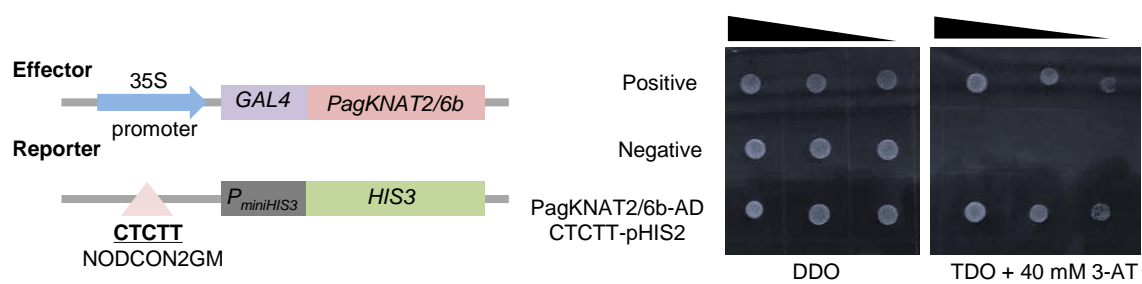

Figure S4

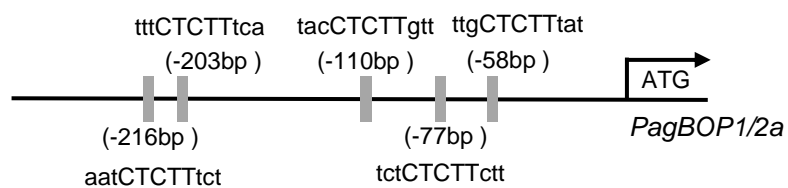

Figure S5

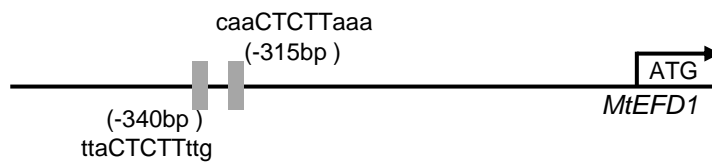

Figure S6

Supplement: Supplementary file 1 [file ijms-23-05581-s001.zip › Supplementary Figures.pdf]
